# Exploring the Causal Effects of Circulating ST2 and Galectin-3 on Heart Failure Risk: A Mendelian Randomization Study

**DOI:** 10.3389/fcvm.2022.868749

**Published:** 2022-04-11

**Authors:** Xizhi Wang, Xingchen Wang, Jun Zhu, Yu Liu, Lenan Zhuang, Zhe Zhang, Danfeng Zhong, Wenbin Zhang, Dongwu Lai

**Affiliations:** ^1^Key Laboratory of Cardiovascular Intervention and Regenerative Medicine of Zhejiang Province, Department of Cardiology, Sir Run Run Shaw Hospital, Zhejiang University School of Medicine, Hangzhou, China; ^2^Department of Cardiac Surgery, Sir Run Run Shaw Hospital, Zhejiang University School of Medicine, Hangzhou, China

**Keywords:** ST2, galectin-3, heart failure, Mendelian randomization, causal association

## Abstract

**Background:**

Heart failure (HF), primarily caused by conditions such as coronary heart disease or cardiomyopathy, is a global health problem with poor prognosis and heavy burden on healthcare systems. As biomarkers of myocardial injury and fibrosis, suppression of tumorigenicity 2 (ST2) and galectin-3 were recommended for prognosis stratification in HF guidelines. However, the causality between these two mediators and HF remains obscure. This study aimed to explore the causal relationship of genetically determined ST2 and galectin-3 with the risk of HF.

**Methods:**

We used the two-sample Mendelian randomization (MR) method, incorporating available genome-wide association summary statistics, to investigate the causal association of ST2 and galectin-3 with HF risk. We applied inverse-variance weighted analysis as the main method of analysis.

**Results:**

In our final MR analysis, 4 single-nucleotide polymorphisms (SNPs) of ST2 and galectin-3, respectively, were identified as valid instrumental variables. Fixed-effect inverse variance weighted (IVW) analysis indicated that genetically predicted ST2 and galectin-3 were not causally associated with HF risk 3. [odds ratio (OR) = 0.9999, 95% confidence interval [CI] = 0.9994–1.0004, *p* = 0.73; OR = 1.0002, 95% CI = 0.9994–1.0010, *p* = 0.60, respectively]. These findings were robust in sensitivity analyses, including MR-Egger regression and leave-one-out analysis.

**Conclusion:**

This MR study provided no evidence for the causal effects of ST2 and galectin-3 on HF risk.

## Introduction

Heart failure (HF) remains a primary public health issue with high rates of hospitalization and mortality (1, 2). HF was caused by many cardiovascular diseases, such as coronary heart disease or cardiomyopathy as a consequence of leading to adverse cardiac remodeling (3, 4). Despite ongoing advances in medications and biomedical devices, pathophysiological progress of HF put patients on a trajectory of poor prognosis (5).

Recent HF guideline recommended biomarkers, such as natriuretic peptides, ST2 and galectin-3, for diagnosis and prognosis in management of HF patients (6). As classical HF biomarkers, BNP (B-type natriuretic peptide) and NT-proBNP (N-terminal pro-B-type natriuretic peptide) are widely used to establish the presence and severity of HF in clinical practice (6). Further, causality assessment of natriuretic peptides and HF indicated that HF therapy targeting natriuretic peptides may work through indirect mechanisms (7).

It is well known that inflammation plays a crucial role in the pathogenesis of HF. As predictive biomarkers for prognosis of HF patients, both soluble ST2 and galectin-3 are extensively involved in inflammatory mechanisms resulting in myocardial fibrosis and adverse cardiac remodeling (8). However, the causal effects of these two mediators on the risk of HF are still not fully elucidated (9).

Mendelian randomization (MR) is a statistical method used to detect and quantify causality using genetic instrumental variables (IVs) as proxies (10). It is not confounded by environmental factors, lifestyle factors, or reverse causation owing to randomly allocated genetic variants (10). The ascendancy of MR is only valid if three core assumptions, as follows, are held: (1) the genetic variant must be associated with the exposure, (2) the genetic variant must not be directly associated with the outcome, and (3) the genetic variant must not be associated with any confounding factor (10).

Thus far, the question of whether ST2 and galectin-3 are involved in the pathogenesis of HF in a causative manner remains obscure. Hence, we aimed to investigate the causality between these two novel biomarkers and HF risk by applying a two-sample MR analysis.

## Materials and Methods

### Study Design

**FIGURE 1:**
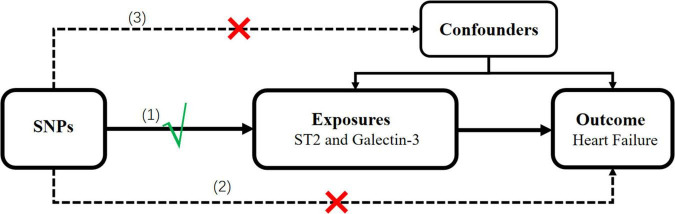
Schematic representation of the MR analysis in this study. Three assumptions of MR analysis are as follows: **(1)** SNPs must be associated with the ST2 and galectin-3, **(2)** SNPs not directly be associated with heart failure, and **(3)** SNPs must not be associated with any confounding factor. MR, Mendelian randomization; SNPs, single-nucleotide polymorphisms.

We selected genetic instruments for circulating ST2 and galectin-3. A two-sample MR study was designed to determine the causal association between these two biomarkers and HF risk based on the available summary-level data from the genome-wide association study (GWAS). The single nucleotide polymorphisms (SNPs) that were selected as IVs were supposed to fulfill the three aforementioned key assumptions; the schematic diagram is presented in Figure 1.

### Data Source

**FIGURE 2:**
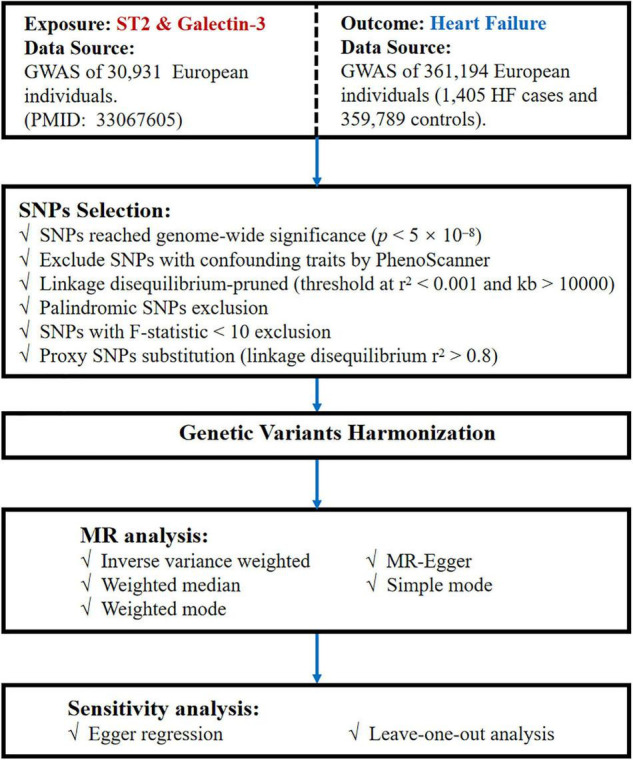
Flow chart of our MR study. MR, Mendelian randomization; GWAS, genome-wide association study; SNPs, single-nucleotide polymorphisms.

Summary statistics data of SNPs related to circulating ST2 and galectin-3 were extracted from the GWAS with a sample size of 30,931 European individuals (11). HF data were obtained by Neale lab analysis of UK Biobank phenotypes,^1^ with a sample size of 361,194 Europeans (1,405 HF cases and 359,789 controls). Adequate patient consent and ethical approval were acquired in the original studies from which data for this study were obtained. A flowchart of the MR analysis performed in the present study is shown in Figure 2.

### Single Nucleotide Polymorphism Validation

First, we selected SNPs associated with ST2 and galectin-3 at a genome-wide significance threshold (*p* < 5 × 10^–8^) from the corresponding datasets to ensure a close relationship existed between the IVs and these two mediator levels. Second, we checked for the, respectively, ST2 and galectin-3-associated SNPs on PhenoScanner^2^ to evaluate whether these SNPs were associated with potential confounders. Next, we excluded those SNPs with confounding traits that may influence the results. Third, given that linkage disequilibrium (LD) is a population-based parameter that measures the non-random association of alleles at different loci, we can apply measures in the MR analysis to explain any correlations of LD. Herein, we clustered the reference GWAS datasets of samples with European, and a similar ancestry is an effective way to assure the independence of all instruments. We also used LD-Link based on the European population to ensure the independence of the selected SNPs by calculating the pairwise-LD. The R software program’s clumping procedure with the “TwoSampleMR” package was performed to automatically drop SNPs with linkage dependence, and for our study, we set the LD threshold at *r*^2^ < 0.001 and kb > 10,000. Moreover, because of the same letters on the forward and reverse strands, palindromic SNPs were deleted to prevent unexpected biases. Subsequently, we extracted information on the genetic associations between the remaining SNPs and HF from the GWAS dataset on HF. When the specified SNP was not available in the HF dataset, a highly correlated SNP (*r*^2^ > 0.8) was selected as proxy. Any SNP directly associated with HF at a genome-wide significance level was excluded. Finally, the estimated variance in ST2 and galectin-3 explained by each SNP and the corresponding F statistics were calculated to evaluate the strength of the IVs (12). To minimize potentially weak instrument bias, we considered an F-statistic of at least 10, as appropriate, for performing a two-sample MR approach.

### Mendelian Randomization Estimates

Primary MR analysis was conducted using the fixed-effect inverse variance weighted (IVW) method, which assumes the absence of invalid genetic instruments (e.g., no heterogeneity, no pleiotropy, and no outlier). Specifically, Wald ratios were estimated for each IV, and the mean IVW of these ratio estimates was calculated as the effect estimate. Finally, results were expressed as odds ratios (ORs) on HF risk for corresponding ST2 and galectin-3. When the MR assumptions were met, the ORs were used as an estimate of the causal effect of the exposure (ST2 and galectin-3) on the outcome (HF).

### Sensitivity Analysis

To further examine the robustness of the effect estimate, the MR-Egger regression and weighted-median sensitivity analysis were performed. In MR-Egger, the intercept estimated the potential horizontal pleiotropic effects in genetic IVs; a value that deviates from the origin may indicate that the IVW estimate is biased. Besides, funnel plots were generated to visually inspect symmetry, which indicated that the causal estimates of weaker variants tended to tilt in one direction, and any deviation may imply potential pleiotropic effects. A weighted-median estimator analysis can provide a consistent valid estimate if more than one-half of the information for the analysis is derived from valid IVs. In addition, we conducted the leave-one-out analysis to assess whether one SNP affected the result. Furthermore, we used Cochran’s Q test to estimate the heterogeneity among the Wald ratios estimated from different genetic variants.

All the analyses were performed in the “TwoSampleMR” package (version 0.5.5) in the software R (version 4.0.3). A *p*-value of < 0.05 was considered statistically significant.

## Results

### Single Nucleotide Polymorphism Selection and Validation

Initially, 9 SNPs of ST2 and 7 SNPs of galectin-3, respectively, were extracted at a genome-wide significant threshold from the corresponding datasets. Then, the SNPs were scanned on PhenoScanner. Three SNPs of ST2 and galectin-3 individually were removed because of their associations with confounding traits, including high cholesterol, hypertension, coronary artery disease and so forth. The detailed information of removed SNPs is presented in Supplementary Table 1. Among them, additional SNPs (rs7604529, rs4311080, and rs6725806) of ST2 and SNPs (rs6650508, rs3742564, rs4363781, rs3825615 and rs62143199) of galectin-3 were dropped due to the LD (*r*^2^ > 0.001, kb < 10000). Moreover, no SNPs were detected with palindromes and the F-statistic value of all SNPs was greater than 10. After the exclusion of these SNPs, the remaining 4 SNPs of ST2 and galectin-3, respectively, were identified as the IVs in our two-sample MR analysis. The detailed information about SNPs for these two exposures included as IVs in our analysis is shown in Table 1.

**TABLE 1:** The characteristics of SNPs and their associations with exposures and outcome.

SNP	Nearest gene	Chr	Position	EA	OA	EAF	F	SNP-exposures association			SNP-HF association		
								Beta	SE	*p*-value	Beta	SE	*p*-value
**ST2**													
rs11603123	KIRREL3	11	126,305,495	A	G	0.032	266	0.3712	0.032	2.61E-47	5.05E-05	0.000399938	0.89949
rs13020553	IL1RL1	2	102,931,826	C	G	0.63	7235	0.6377	0.0088	6.89E-1635	−8.08E-05	0.000147981	0.585092
rs2460382	MGAT5	2	135,014,116	A	G	0.77	56	−0.0714	0.011	1.31E-14	0.00027079	0.000172428	0.116312
rs672806	DCPS	11	126,188,405	A	G	0.4	149	0.0998	0.0098	1.43E-34	0.000210482	0.000147057	0.152346
**Galectin-3**													
rs3735080	GIMAP7	7	150,217,309	T	C	0.24	86	0.087	0.011	8.41E-21	−3.48E-05	0.00017118	0.839049
rs62143206	NLRP12	19	54,326,212	T	G	0.21	74	0.0849	0.011	1.19E-19	−0.000162714	0.000176406	0.35633
rs76480089	ATG14	14	55,832,612	A	G	0.92	1885	0.6247	0.015	1.28E-512	0.000229768	0.000259548	0.376016
rs812936	FUT3	19	5,844,649	A	G	0.8	85	0.0925	0.012	2.59E-20	−9.77E-05	0.000188528	0.604367

*SNPs, single-nucleotide polymorphisms; HF, heart failure; Chr, chromosome; EA, effect allele; OA, other allele; EAF, effect allele frequency; SE, standard error.*

### Analysis Using the Two-Sample Mendelian Randomization

**FIGURE 3:**
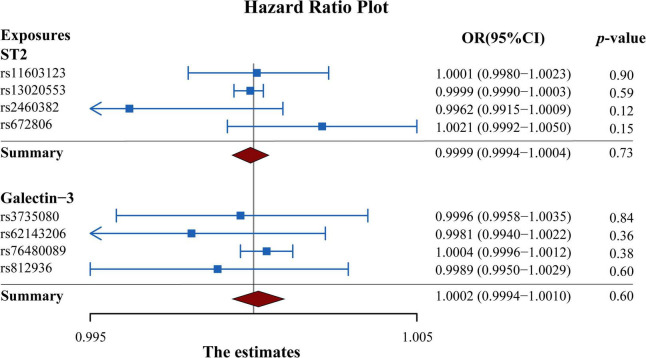
Forest plot summarizing the overall MR estimates of SNP specificity and the causal effect on heart failure. Estimated odds ratios represent the effect per standard deviation increase in circulating ST2 and galectin-3 levels on heart failure, respectively. Results were obtained from the fix-effect IVW method. Squares represent the estimated ORs, and horizontal lines represent the 95% CI of the ORs. MR, Mendelian randomization; SNP, single-nucleotide polymorphism; IVW, inverse variance weighted; OR, odds ratio; CI, confidence interval.

**FIGURE 4:**
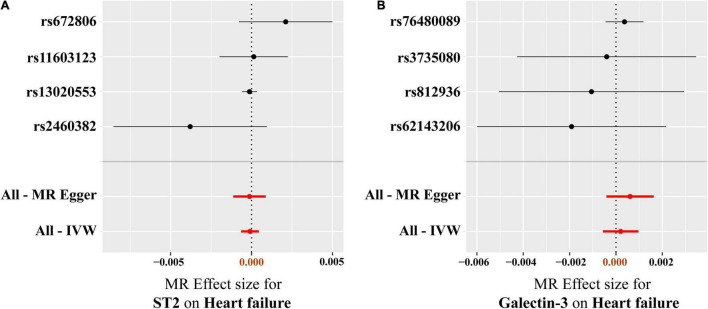
MR-Egger and fixed-effect IVW analysis of the causal association between circulating ST2 and galectin-3 levels and heart failure risk. **(A)** ST2, **(B)** galectin-3. Forest plots reflect the causal effect of individual SNPs on heart failure. Black dots represent the estimated betas, and horizontal lines represent the 95% CI of the betas. IVW, inverse variance weighted; SNPs, single-nucleotide polymorphisms; CI, confidence interval; MR, Mendelian randomization.

As presented in Figure 3, the results of the MR analysis were expressed as odds ratios (ORs) of HF per standard deviation (SD) increase in circulating ST2 and galectin-3, indicating that neither genetically predicted ST2 nor galectin-3 were causally associated with the risk of HF (*p* > 0.05). Remarkably, the fixed-IVW method and other statistical methods of MR analysis showed similar results (Figure 4 and Supplementary Tables 2, 3). Scatter plots of the potential effects of both ST2 and galectin-3-associated SNPs on HF are shown in Supplementary Figure 1.

### Sensitivity Analysis

**FIGURE 5:**
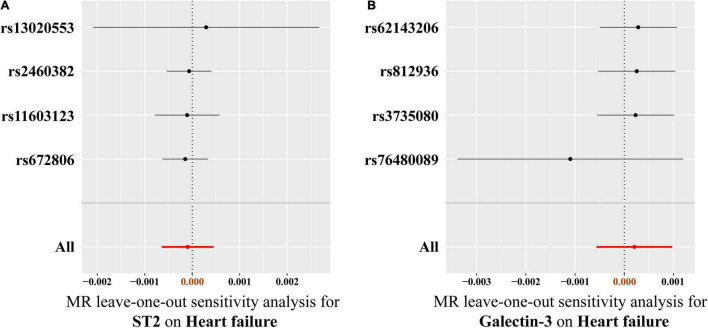
Leave-one-out analysis of the causal association between circulating ST2 and galectin-3 levels and heart failure risk. **(A)** ST2, **(B)** galectin-3. Each SNP was excluded sequentially and the MR estimate effect was recalculated. Visually, the leave-one-out analysis plot illustrated that the results were not driven by any SNPs. SNP, single-nucleotide polymorphism; MR, Mendelian randomization.

Results of heterogeneity and directional horizontal pleiotropy bias are shown in Supplementary Tables 4, 5, respectively. There was no heterogeneity in ST2 and galectin-3 (All *p* > 0.05) (Supplementary Table 4). The MR-Egger regression and the appearance of the funnel plots showed that there was a low likelihood of horizontal pleiotropy for our estimations (Both *p* for MR-Egger intercept > 0.05) (Supplementary Table 5 and Supplementary Figure 2). No outlier was detected in the leave-one-out analysis, and the results illustrated that the overall estimate was not driven by any SNPs (Figure 5). Additionally, we performed MR analysis of all SNPs that included SNPs dropped due to LD or potential pleiotropy. The results were also negative, which showed no significant causal association of circulating ST2 and galectin-3 on HF (Supplementary Table 6).

## Discussion

In this study, we examined the causal effects of circulating ST2 and galectin-3 on HF risk using MR analysis. Collectively, we did not find evidence that genetically determined ST2 and galectin-3 are causally associated with the risk of HF. To the best of our knowledge, this is the first study concerning the issue by MR analysis.

It is well known that inflammation plays a critical role in the pathophysiological processes of HF (3, 13). As inflammatory mediators involved in myocardial injury and fibrosis, soluble ST2 and galectin-3 were recommended for additive risk stratification of HF patients in the latest guideline (class II b, evidence level B) (6).

ST2 is a member of the interleukin (IL)-1 receptor family. Two isoforms of ST2, transmembrane receptor (ST2L) and soluble decoy receptor (sST2), are both bound to IL-33 (14). IL-33 could be activated by mechanical stress or inflammatory signals and released from cells (15). It acts as an “alarmin” on neighboring or immune cells expressing the ST2 receptor (16). Once released, IL-33 would activate the ST2L on nearby cells. sST2 could be secreted into the circulation by endothelial and various immune cells upon stimulation like IL-33 (17). IL-33 was thought to exert beneficial actions via ST2L that are related to cardiac repair or attenuation of adverse cardiac remodeling (15). Acting as a soluble truncated form of ST2L, sST2 functions as a decoy receptor for IL-33 and thereby attenuates beneficial effects of IL-33/ST2L signaling in the context of the heart (18). Previous clinical and experimental studies showed a strong correlation between ST2 and HF (19). Clinical evidence suggested that elevation of baseline sST2 was significantly associated with worsening HF (20, 21). In pressure-overload murine model, deletion of ST2 enhanced myocardial fibrosis and cardiac hypertrophy, suggesting a potential role for IL-33/ST2 signaling axis in cardiac remodeling under mechanical overload (22).

As a member of the β-galactoside-binding protein family, galectin-3 stimulates myofibroblasts proliferation and procollagen deposition, which lead to cardiac fibrosis and hypertrophy (23). In past years, a growing body of literature emerged on galectin-3 in HF patients. However, the causal relationship between galectin-3 and HF risk remains obscure. In clinical studies, such as Framingham Offspring Cohort study and Val-HeFT study, higher level of serum galectin-3 was associated with increased risk for incidence, hospitalization and all-cause mortality of HF (24, 25). In rat HF model, elevated galectin-3 level marked macrophages activation and contributed to cardiac dysfunction (26). Besides, attenuated cardiac fibrosis, left-ventricular dysfunction were also observed in genetic galectin-3 knockout mouse (27).

Nevertheless, some other studies showed different results. In RELAX-AHF study, enrolled 1,161 acute HF patients, galectin-3 levels remained stable over time and presented no independent relationship with cardiovascular mortality at 180 days (28). Likewise, no relationship was found between galectin-3 values and mortality events or heart transplantation in a study of chronic HF patients (29). In the mice myocardial infarction model, genetic deletion of galectin-3 altered the temporal evolution of macrophage infiltration and wound healing, which affected cardiac remodeling and function (30).

Considering to the aspect of HF risk in general population, our findings are not contradictory with previous studies. To some extent, circulating ST2 and galectin-3 are not cardiac-specific biomarkers (9). Concentration variation of these two mediators in serum is not parallel with that in myocardium. Furthermore, observational results are usually subjected to confounding factors (31). In the present study, from a genetic perspective, we found no causal effect of circulating ST2 and galectin-3 on HF risk, indicating that increased ST2 and galectin-3 in HF patients may be an epiphenomenon, rather than major driving factors in the pathogenesis of HF.

Randomized controlled trial is a powerful approach for proving hypothesis in epidemiological research. However, it requires rigorous design, strictly enroll/exclude standards and expensive cost. By using genetic variants as instruments of exposure, MR analysis mimics the randomization in clinical trials. MR analysis could overcome the potential impact of confounding factors and minimize the bias in causal effect estimates as much as possible (32). Additionally, sensitivity analysis appeared that the causal estimates were robust in our study.

There are some limitations in our study. First, the utilized datasets were obtained from individuals of European ancestry and were not stratified by the severity of HF. Further work is warranted to investigate whether similar findings are displayed in different ethnicities, subtypes and severity of HF patients. Second, because of a lack of data on exposure and outcomes, bidirectional MR analysis was not performed to study the reverse causation of HF on circulating ST2 and galectin-3. Third, we did not explore the causality between IL-33 and HF due to the lack of available data from the GWAS. Therefore, these questions deserve further study. Fourth, given the lifetime effect of SNPs, our research assumes that these mediators act on HF for a long time, which differs from the situation of clinical practice. Moreover, we appraised the causal association without consideration of environmental factors. Nevertheless, this study delivered some valuable clues into the pathophysiologic progress of HF, and we hope that our findings would promote the therapeutic strategy exploration of HF.

## Conclusion

This study provided no evidence for causative effects of circulating ST2 and galectin-3 on the risk of HF incidence, indicating that these two mediators may not be major driving factors in the pathogenesis of HF. However, further studies are needed to validate our findings.

## Data Availability Statement

The original contributions presented in the study are included in the article/Supplementary Material, further inquiries can be directed to the corresponding author/s.

## Author Contributions

XZW, JZ, YL, and ZZ collected and interpreted the data. XZW and XCW analyzed the data. XZW, XCW, LZ, and DZ generated the figures and wrote the manuscript. DL and WZ designed the study and revised the manuscript. All authors approved the final manuscript.

## Conflict of Interest

The authors declare that the research was conducted in the absence of any commercial or financial relationships that could be construed as a potential conflict of interest.

## Publisher’s Note

All claims expressed in this article are solely those of the authors and do not necessarily represent those of their affiliated organizations, or those of the publisher, the editors and the reviewers. Any product that may be evaluated in this article, or claim that may be made by its manufacturer, is not guaranteed or endorsed by the publisher.

## Footnotes

1
http://www.nealelab.is/uk-biobank

2
http://www.phenoscanner.medschl.cam.ac.uk/

## Abbreviations

HF: heart failure
MR: Mendelian randomization
SNP: single-nucleotide polymorphism
IV: instrumental variable
GWAS: genome-wide association study
IL: interleukin.
