# Supplementary material for: Exploring the Causal Effects of Circulating ST2 and Galectin-3 on Heart Failure Risk: A Mendelian Randomization Study
Source: Front Cardiovasc Med. 2022 Apr 11;9:868749. doi: 10.3389/fcvm.2022.868749 (PMC9037587; doi:10.3389/fcvm.2022.868749)
Supplement: Supplementary file 1 [file Data_Sheet_1.PDF]

# *Supplementary Material*

## 1 Supplementary Figures

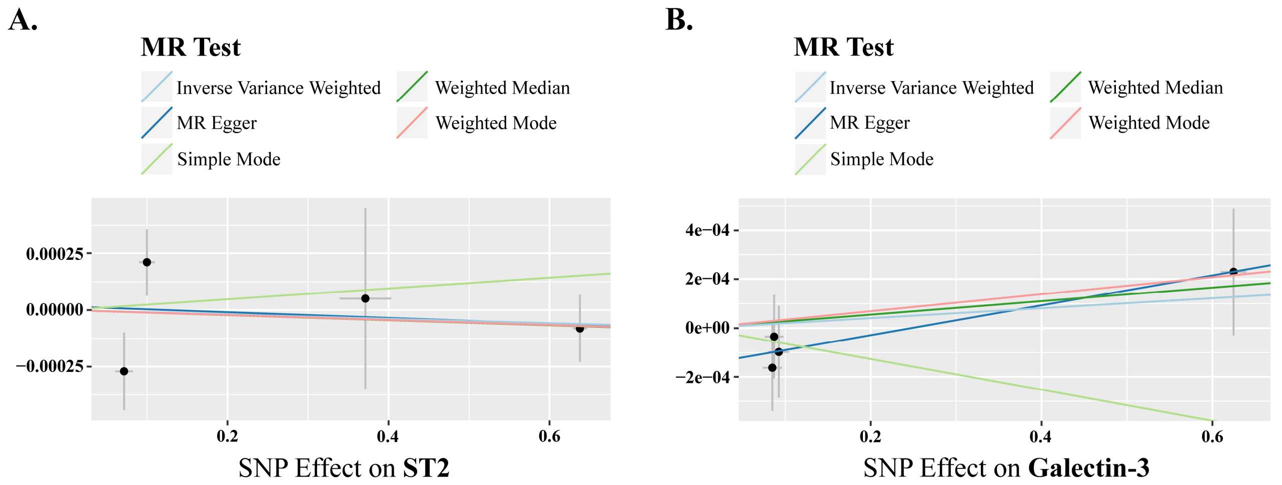


**Supplementary Figure 1. Scatter plot visualizing the SNPs related to ST2 and galectin-3 levels.** **(A)** ST2, **(B)** Galectin-3. Each black dot represents an SNP, plotted by SNP estimates at circulating ST2 and galectin-3 levels and SNP estimates at the risk of HF individuals with standard error bars. The slope of the line corresponds to the causal estimates made using five different methods. SNPs, single-nucleotide polymorphisms; HF, heart failure; MR, Mendelian randomization.

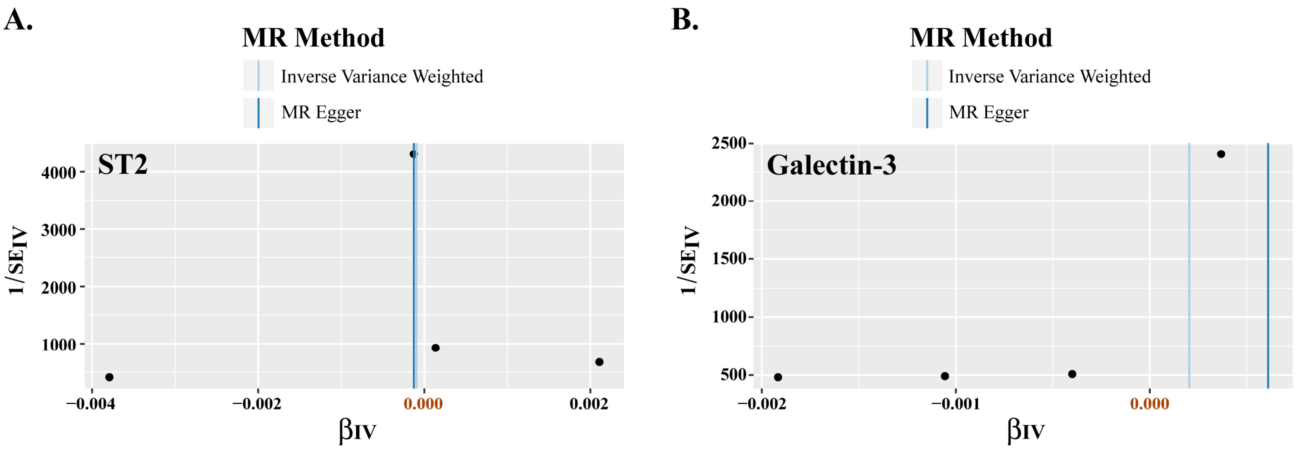


**Supplementary Figure 2. Funnel plot visualizing the SNPs related to ST2 and galectin-3 levels. (A)** ST2, **(B)** Galectin-3. Each black dot represented an SNP, with the overall estimate obtained from two methods. Funnel plots were used to visually examine symmetry, which can roughly detect whether causal estimates of weak variants tended to skew in one direction. SNPs, single-nucleotide polymorphisms; MR, Mendelian randomization.

## 2 Supplementary Tables

**Supplementary Table 1. Association of SNPs with confounding traits at a genome-wide significance level ($p < 5 \times 10^{-8}$).**

| SNP | Nearest gene | Chr | EA | OA | EAF | Confounding traits |
|---|---|---|---|---|---|---|
| **ST2** | | | | | | |
| rs186021206 | ASGR1 | 17 | A | G | 0.0088 | Self-reported high cholesterol |
| rs35518360 | SLC39A8 | 4 | A | T | 0.93 | Body mass index; Hip circumference; Diastolic blood pressure |
| rs635634 | ABO | 9 | T | C | 0.2 | LDL cholesterol; Total cholesterol; Coronary artery disease |
| **Galectin-3** | | | | | | |
| rs59379014 | ST3GAL4 | 11 | T | C | 0.077 | Low density lipoprotein; High density lipoprotein; Coronary artery disease |
| rs7979473 | HNF1A | 12 | A | G | 0.4 | C-reactive protein; Coronary artery disease; High cholesterol |
| rs660745 | MAMSTR | 19 | T | C | 0.52 | High cholesterol; Hypertension; Alcohol intake frequency |

SNPs, single-nucleotide polymorphisms; Chr, chromosome; EA, effect allele; OA, other allele; EAF, effect allele frequency.

**Supplementary Table 2. Different statistical methods for MR analysis evaluating the causal association between exposures and outcome.**

| Methods | ST2 | | | | Galectin-3 | | | |
|---|---|---|---|---|---|---|---|---|
| | nSNP | Beta | SE | *p* | nSNP | Beta | SE | *p* |
| MR-Egger | 4 | -0.000125817 | 0.000511786 | 0.828734498 | 4 | 0.000609924 | 0.000520099 | 0.361682015 |
| Weighted median | 4 | -0.000111127 | 0.000218912 | 0.611711407 | 4 | 0.000275672 | 0.000402713 | 0.493636564 |
| Inverse variance weighted | 4 | -9.54E-05 | 0.000277834 | 0.731269768 | 4 | 0.000203772 | 0.000391519 | 0.602739102 |
| Simple mode | 4 | 0.000238001 | 0.000597494 | 0.717040846 | 4 | -0.000633557 | 0.00105196 | 0.589475841 |
| Weighted mode | 4 | -0.000109511 | 0.000234136 | 0.671862055 | 4 | 0.000345983 | 0.00044158 | 0.490491803 |

MR, Mendelian randomization; nSNP, number of single-nucleotide polymorphism for final analysis; SE, standard error.

**Supplementary Table 3. MR analysis results of a single SNP.**

| **Traits** | | **Beta** | **SE** | ***p*** |
|---|---|---|---|---|
| **ST2** | | | | |
| | rs672806 | 0.002109038 | 0.001473517 | 0.152345587 |
| | rs11603123 | 0.000136084 | 0.001077419 | 0.899490239 |
| | rs13020553 | -0.000126693 | 0.000232054 | 0.585092071 |
| | rs2460382 | -0.003792577 | 0.002414958 | 0.116309855 |
| | All - MR Egger | -0.000125817 | 0.000511786 | 0.828734498 |
| | All - Inverse variance weighted | -9.54E-05 | 0.000277834 | 0.731269768 |
| **Galectin-3** | | | | |
| | rs76480089 | 0.000367805 | 0.000415476 | 0.376015302 |
| | rs3735080 | -0.000399637 | 0.001967586 | 0.839048918 |
| | rs812936 | -0.001056019 | 0.002038141 | 0.604368357 |
| | rs62143206 | -0.001916537 | 0.002077809 | 0.356328525 |
| | All - MR Egger | 0.000609924 | 0.000520099 | 0.361682015 |
| | All - Inverse variance weighted | 0.000203772 | 0.000391519 | 0.602739102 |

MR, Mendelian randomization; SNP, single-nucleotide polymorphism; SE, standard error.

**Supplementary Table 4. Heterogeneity test for each trait.**

| **Method** | **ST2** | | **Galectin-3** | |
|---|---|---|---|---|
| | **Q** | **Q_*p*val** | **Q** | **Q_*p*val** |
| **MR-Egger** | 4.63167358 | 0.098683572 | 0.265999663 | 0.87546524 |
| **Inverse variance weighted** | 4.646280627 | 0.199607662 | 1.673307263 | 0.642884416 |

**Supplementary Table 5. Summary for directional horizontal pleiotropy tests.**

| **Traits** | **ST2** | | **Galectin-3** | |
|---|---|---|---|---|
| | Egger intercept | *p* | Egger intercept | *p* |
| **Egger Regression** | 1.59E-05 | 0.943930265 | -0.000150777 | 0.357328789 |

**Supplementary Table 6. Different statistical methods for MR analysis evaluating the causal association between exposures and outcome that used all extracted SNPs.**

| **Methods** | **ST2** | | | | **Galectin-3** | | | |
|---|---|---|---|---|---|---|---|---|
| | nSNP | Beta | SE | *p* | nSNP | Beta | SE | *p* |
| MR-Egger | 20 | -0.00003 | 0.000295 | 0.928866 | 11 | 0.000306 | 0.00044 | 0.50445 |
| Weighted median | 20 | -0.00014 | 0.000219 | 0.517754 | 11 | 0.000357 | 0.000385 | 0.354314 |
| Inverse variance weighted | 20 | -0.00024 | 0.00019 | 0.206033 | 11 | 0.000252 | 0.000302 | 0.402936 |
| Simple mode | 20 | -0.00032 | 0.000467 | 0.505895 | 11 | 0.000389 | 0.000764 | 0.621783 |
| Weighted mode | 20 | -0.00018 | 0.000214 | 0.422247 | 11 | 0.000389 | 0.000393 | 0.345567 |

MR, Mendelian randomization; nSNP, number of single-nucleotide polymorphism that used all extracted SNPs; SE, standard error.
